# Case report: atypical presentation of acute disseminated encephalomyelitis after tick-borne encephalitis

**DOI:** 10.1093/omcr/omad137

**Published:** 2023-12-19

**Authors:** Adam Garkowski, Jakub Okrzeja, Bożena Kubas, Piotr Czupryna, Joanna Zajkowska, Adam Kuchta, Anna Moniuszko-Malinowska

**Affiliations:** Department of Radiology, Medical University of Białystok, Białystok, Poland; Medical University of Białystok, Białystok, Poland; Department of Radiology, Medical University of Białystok, Białystok, Poland; Department of Infectious Diseases and Neuroinfections, Medical University of Białystok, Białystok, Poland; Department of Infectious Diseases and Neuroinfections, Medical University of Białystok, Białystok, Poland; J. Śniadecki Provincial Hospital of Białystok, Białystok, Poland; Department of Infectious Diseases and Neuroinfections, Medical University of Białystok, Białystok, Poland

## Abstract

Acute disseminated encephalomyelitis (ADEM) is a rare and an immune- mediated inflammatory illness of the central nervous system that normally demonstrates as a monophasic disorder connected with multifocal neurologic symptoms. Herein, we report atypical presentation of ADEM presenting as single lesions in a middle-aged woman after tick-borne encephalitis.

## INTRODUCTION

Acute disseminated encephalomyelitis (ADEM) is a rare and an immune- mediated inflammatory disease of the central nervous system that typically manifests as a monophasic disorder associated with multifocal neurologic symptoms. This condition is often preceded by viral infection or recent vaccination [1]. ADEM is most commonly seen in pediatric age group and young adults, but very few cases have been reported of older adults with ADEM [2]. Herein, we report atypical presentation of ADEM presenting as single lesions in a middle-aged woman after tick-borne encephalitis.

## CASE REPORT

A 51-year-old woman with a history of arterial hypertension and tick bite (3 weeks before admission to the hospital), was referred to the Department of Infectious Diseases and Neuroinfection in Białystok (Poland) with a 3 days history of headache, and fever (38°C). On admission, physical examination revealed a temperature of 38.5°C, and nuchal rigidity. The neurologic examination of the patient was found completely normal. Brain CT scan did not reveal any abnormalities. In laboratory tests, increased inflammation parameters were observed: a white blood cell count (15.52×10^9^/l), and C-reactive protein concentration (34.6 mg/dl). The CSF examination revealed inflammatory features: pleocytosis—139 cells/μl (neutrophils—36%, lymphocytes—59%, monocytes—5%), total protein—90.1 mg/dl, albumin—66.2 mg/dl, and glucose—54 mg/dl. Serum and CSF anti-tick-borne encephalitis virus IgM antibodies (serum—75 U/ml, CSF—16 U/ml), and IgG (serum—347 U/ml, CSF—269 U/ml) were positive (ELISA test) (serum and CSF cut-off values: 15 U/ml for IgM, 28 U/ml for IgG). The diagnosis of tick-borne encephalitis (TBE) was made. Treatment with 20% intravenous mannitol (3 × 100 ml), and oral vinpocetine was started (daily dosage of 15 mg). The patient was discharged in good general condition after 14 days of hospitalization.

**Figure 1:**
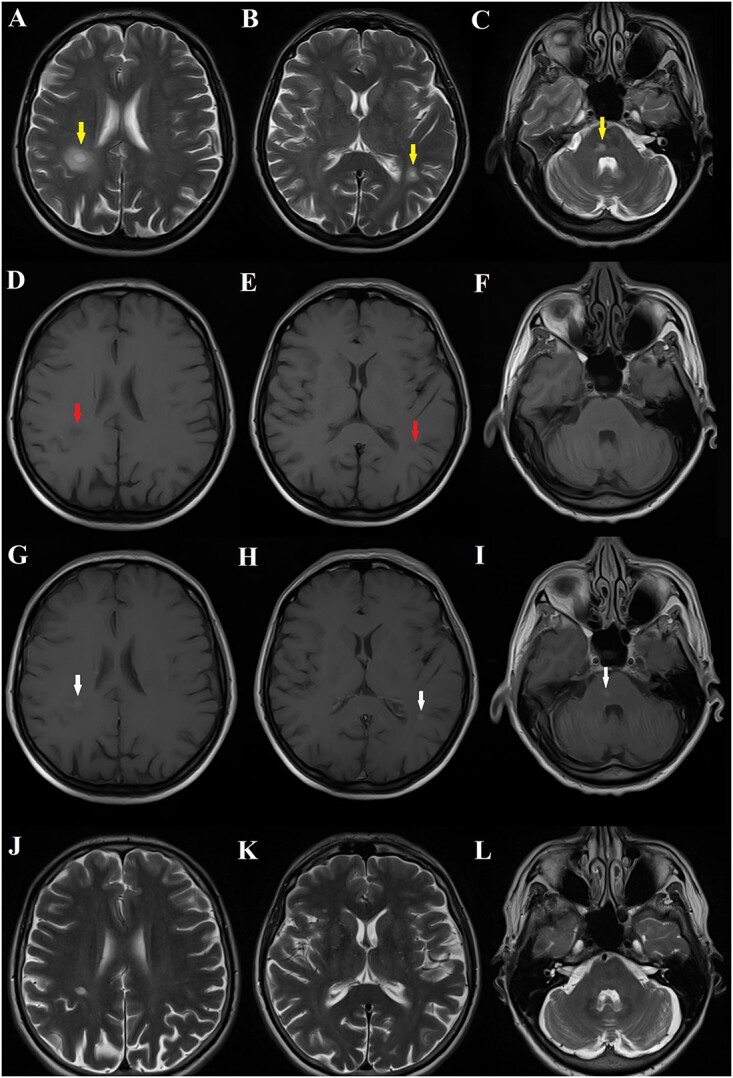
MRI of the brain of a 51-year old woman with a history of tick-borne encephalitis. Axial T2-weighted images (**A, B, C**) depict high-signal lesions with surrounding edema in the subcortical white matter of the right and left parietal lobe and in the pons. On axial pre-contrast T1-weighted images (**D, E, F**) two lesions appeared as a low signal intensity and the lesion located in the pons was imperceptible. On axial contrast enhanced T1-weighted images (**G, H, I**) all lesions showed contrast enhancement. Follow-up MRI demonstrates no new lesions and that previously demonstrated lesions markedly reduced in size, with some residual abnormal T2 signal in the cerebral hemispheres (**J, K, L**).

At follow-up visit after 1 months, the patient complained about a two week history of moderate headaches and gait disturbance with left-sided weakness. The neurologic examination showed mild left-sided hemiparesis. Brain MRI revealed single hyperintense contrast-enhancing lesions in the right and left hemisphere, and in the pons (Fig. 1). Spine MRI was normal. Serological examinations, which included HSV, zoster, HIV, and syphilis, were negative. CSF examination was unremarkable. The absence of oligoclonal bands at follow-up CSF analysis, along with the history of TBE earlier suggested the diagnosis of multiple sclerosis unlikely, and led us to suggest the diagnosis of ADEM. Administration of steroids was started and the patient significantly improved, and there were no neurological deficits.

The patient returned for further imaging and repeated MRI after 3, 12, and 24 months showed significant lesions regression enabled us to confirm that the lesions were not neoplastic, and imaging showed no new lesions in other areas of the brain excluding multiple sclerosis (Fig. 1). What is more, during follow-up MRIs, the patient showed no features of encephalopathy or other neurological deficits.

## DISCUSSION

Herein, we report the case of probable ADEM in which imaging features demonstrated a single lesions in the brain. Additionally, to our knowledge, this is the first described case of ADEM after TBE. TBE is a potentially fatal disease caused by tick-borne encephalitis virus (TBEV), involving the central nervous system and affecting humans in Europe and Asia. TBEV is a member of the genus *Flavivirus*, within the family *Flaviviridae* and is transmitted by the ticks *Ixodes ricinus* and *Ixodes persulcatus* [3, 4].

Furthermore, TBE is an illness which might present as meningitis, meningoencephalitis and meningoencephalomyelitis. European TBEV subtype normally produces biphasic course and tends to be less severe, although it could be mortal in 1%–2% of cases [5]. There are a lot of risk agents that influence the severity of TBE, e.g. individual’s age, immunosuppression and concomitant illnesses [6]. Many factors contribute to the suppression of the immune system: HIV infection, organ transplantation, therapy of autoimmune diseases, malignancies and cancer treatment [7].

ADEM is an autoimmune, usually a monophasic demyelinating disease of the central nervous system, commonly induced by viral or bacterial infections or vaccination after a lag time of a few days to several weeks. During the acute phase of ADEM, proinflammatory factors and antibodies targeting gangliosides are responsible for demyelinating injury to white matter in the cerebrum and spinal cord [8–11]. Brain lesions associated with this condition are typically multiple, bilateral but asymmetric, indicating demyelination [8–11]. Single lesions are rarer, and atypical for ADEM [12]. On MRI, the lesions of ADEM are hyperintense on T2-weighted and FLAIR images. Punctate, ring or arc gadolinium enhancement may be also present, however absence of enhancement does not rule out the diagnosis [8]. What is more, ADEM is featured by encephalopathy and non-specific, heterogeneous neurological disorders depending on the area of the demyelinated changes [10].

## CONCLUSIONS

This case report describes a presentation of ADEM presenting as single lesions in a middle-aged woman after TBE. The occurrence of ADEM after TBE is very rare condition (especially in this age group). The purpose of this case is to present a case about an uncommon neurologic disease, which every clinician may meet in clinical practice.

## CONFLICT OF INTEREST STATEMENT

No conflicts of interest.

## FUNDING

The authors have not received any outside funding.

## ETHICAL APPROVAL

Ethical approval was not required for this study.

## CONSENT

Informed consent has been obtained.

## GUARANTOR

Adam Garkowski.
